# Characteristics of *pncA *mutations in multidrug-resistant tuberculosis in Taiwan

**DOI:** 10.1186/1471-2334-11-240

**Published:** 2011-09-12

**Authors:** Yu-Chi Chiu, Shiang-Fen Huang, Kwok-Woon Yu, Yu-Chin Lee, Jia-Yih Feng, Wei-Juin Su

**Affiliations:** 1 Department of Chest Medicine, Taipei Veterans General Hospital, Shih-Pai Rd., Taipei 112, Taiwan, ROC; 2 Institute of Clinical Medicine, National Yang-Ming University, Linong Street, Taipei 112, Taiwan, ROC; 3 School of Medicine, National Yang-Ming University, Linong Street, Taipei 112, Taiwan, ROC; 4 Section of Microbiology, Department of Laboratory Medicine, Taipei Veterans General Hospital, Shih-Pai Rd., Taipei 112, Taiwan, ROC

## Abstract

**Background:**

Pyrazinamide (PZA) is an important first-line drug in multidrug-resistant tuberculosis (MDRTB) treatment. However, the unreliable results obtained from traditional susceptibility testing limits its usefulness in clinical settings. The detection of *pncA *gene mutations is a potential surrogate of PZA susceptibility testing, especially in MDRTB isolates. The impact of genotypes of *M. tuberculosis *in *pncA *gene mutations also remains to be clarified.

**Methods:**

MDRTB isolates were collected from six hospitals in Taiwan from January 2007 to December 2009. *pncA *gene sequencing, pyrazinamidase activity testing, and spoligotyping were performed on all of the isolates. PZA susceptibility was determined by the BACTEC MGIT 960 PZA method. The sensitivity and specificity of *pncA *gene analysis were estimated based on the results of PZA susceptibility testing.

**Results:**

A total of 66 MDRTB isolates, including 37 Beijing and 29 non-Beijing strains, were included for analysis. Among these isolates, 36 (54.5%) were PZA-resistant and 30 (45.5%) were PZA-susceptible. The PZA-resistant isolates were more likely to have concomitant resistance to ethambutol and streptomycin. Thirty-seven mutation types out of 30 isolates were identified in the *pncA *gene, and most of them were point mutations. The sensitivities of *pncA *gene sequencing for PZA susceptibility in overall isolates, Beijing and non-Beijing strains were 80.6%, 76.2%, and 86.7% respectively, and the specificities were 96.7%, 93.8%, and 100% respectively.

**Conclusions:**

More than half of the MDRTB isolates in this study are PZA-resistant. Analysis of *pncA *gene mutations helped to identify PZA-susceptible MDRTB isolates, especially in non-Beijing strains.

## Background

Despite the recent advances in management, tuberculosis (TB) remains a leading cause of mortality and morbidity worldwide. The emergence of multidrug-resistant tuberculosis (MDRTB) further constitutes a serious threat to the control of TB. In Taiwan, the incidence of TB was 63.2/100,000 population in 2007 and 62.0/100,000 population in 2008 (1). The Taiwan drug resistance surveillance program revealed that the combined drug resistance rates in 2005 were 10.1% for isoniazid (INH), 6.2% for rifampicin (RIF), and 4.0% for MDRTB (2). Among the various genotypes of *Mycobacterium tuberculosis *(MTB), the Beijing genotype is the dominant strain in Taiwan (3, 4).

Pyrazinamide (PZA) is an important first-line anti-tuberculosis (anti-TB) drug that is used in short-course chemotherapy and is one of the cornerstone drugs in the treatment of MDRTB (5). Through pyrazinamidase (PZase) which is constitutively expressed in *Mycobacterium tuberculosis *(MTB), PZA is hydrolyzed to toxic pyrazinoic acid (POA) intracellularly (6, 7). Although the exact mechanism is unknown, it has been postulated that POA exerts an inhibitory effect on cellular metabolism in acidic conditions (8-10). The PZase enzyme is encoded by the *pncA *gene (11). Mutations in the *pncA *gene may cause a reduction in PZase activity which may be the major mechanism of PZA resistance in MTB (12, 13). Previous reports have well characterized the mutations of the *pncA *gene in PZA-resistant MTB isolates, however the correlation varies between different geographical areas (14-17). A great diversity of *pncA *gene mutations has also been described, including missense mutations, one or more base insertions or deletions, and complete deletion (12, 13, 18). Meanwhile, a substantial proportion of resistant isolates have been found to have intact PZase activity without *pncA *gene mutations, suggesting an alternative mechanism of PZA resistance.

Due to the inhibitory effect of low pH on in vitro growth of MTB, conventional PZA drug susceptibility testing on a solid medium is of limited value and not routinely done in many areas (19). Considering the unique bactericidal effect of PZA among first-line anti-TB drugs, it is important to identify PZA resistance in clinical practice, especially in dealing with MDRTB cases. The purpose of the present study was to identify the characteristics of *pncA *mutations in clinical MDRTB isolates, and evaluate the effectiveness of *pncA *gene analysis in identifying PZA-susceptible isolates. The impact of genotyping in PZA resistance was also investigated.

## Methods

### Mycobacterial isolates

This study was conducted in six hospitals in Taiwan, including five referral medical centers and one regional hospital that specialize in pulmonary diseases. MDRTB clinical isolates were collected from newly diagnosed tuberculosis patients from January 2007 to December 2009. The demographic profiles and clinical characteristics of the patients were obtained from medical records, and the chest radiograms were interpreted by the in-charge doctors of each hospital. The institutional review boards of all six hospitals approved the study and informed consent was obtained from each patient before enrollment.

### Drug susceptibility testing (DST) and PZase assay

The drug susceptibilities of isoniazid and rifampicin were performed by the proportion method and their critical concentrations for resistance were as follows: isoniazid, 0.2 μg/ml and 1.0 μg/ml; rifampicin, 1.0 μg/ml and 5.0 μg/ml (20). MDRTB isolates were defined as those resistant to low drug concentration levels in both isoniazid and rifampicin. The susceptibility of PZA was assessed by the non-radiometric BACTEC Mycobacteria Growth Indicator Tube (MGIT) 960 method (BD Biosciences, Sparks, MD, USA). The recommended critical concentration of 100 μg/ml PZA was used to discriminate between PZA-susceptible and PZA-resistant isolates. The PZase activity test was performed according to Wayne's procedure (21).

### DNA extraction, *pncA *gene sequencing

Genomic DNA was extracted from Middlebrook 7H11 cultures as described previously (22). Entire *pncA *genes and 82 bp of an upstream putative regulatory sequence were amplified by polymerase chain reaction (PCR) with forward (P1) and reverse (P6) primers as described previously (23). Briefly, the PCR mixture (100 μL) contained ~1 ng of DNA template, final concentrations of 1.0 μM of each set of primers, 200 μM deoxynucleoside triphosphate (dATP, dCTP, dGTP and dTTP; Pharmacia, Uppsala, Sweden) and 10 μL Taq buffer and 5.0 U/μL Taq polymerase (Gibco-Bethesda Research Laboratories). Amplification was performed for 40 cycles (1 min at 94°C, 1 min at 56°C and 1 min at 72°C) by a GeneAmp PCR system 9700 thermocycler (Perkin Elmer, Applied Biosystems, CA). To determine the *pncA *sequence, PCR products were purified with a QIAquick PCR purification kit (Qiagen GmbH, Hilden, Germany). The PCR products were directly sequenced using an ABI 377 automatic DNA sequencer (Perkin Elmer, Applied Biosystems, CA).

### Genotyping

All clinical isolates were genotyped by a commercial spoligotyping kit (Isogen Bioscience B.V., Maarssen, Netherlands). The "Beijing strain" was defined as deletion from spacer 1 to spacer 34 in the direct repeat region and the presence of (at least 3) spacers 35-43.

### Data analysis

Comparisons of demographic and clinical characteristics were done using the chi-square test or Fisher's exact test for categorical variables, and the two-tailed independent *t *test for continuous variables. Binary logistic regression analysis was performed to determine the independent variables, and odds ratios with their 95% confidence intervals were presented. The sensitivity, specificity, positive predictive value (PPV) and negative predictive value (NPV) of the *pncA *gene mutations and PZase activity test were calculated according to the PZA susceptibility results. Significance was defined as p < 0.05 (two-tailed). Statistical analysis was performed using a statistical software package (SPSS version 17.0, SPSS Inc., Chicago, IL, USA).

## Results

### Patient characteristics

During the study period, a total of 66 MDRTB isolates were collected from newly diagnosed tuberculosis patients in six hospitals in Taiwan. Among these MDRTB isolates, 36 (54.5%) were resistant to PZA and 30 (45.5%) were susceptible to PZA according to the DST results assessed by the MGIT 960 method. The demographic data of these patients are shown in Table 1. MDRTB isolates with concomitant PZA resistance were more likely to be sputum smear negative at the diagnosis of tuberculosis (52.8% vs. 26.7%, p = 0.032), and more likely to be associated with ethambutol (58.3% vs. 33.3%, p = 0.043) and streptomycin resistance (66.7% vs. 36.7%, p = 0.015). Other demographic profiles, including age, gender, previous anti-TB treatment, smear positivity, and the presence of cavities in radiographs, were comparable between patients infected with PZA-resistant or susceptible MDRTB isolates. Of these MDRTB isolates, 37 (56.1%) were Beijing and 29 (43.9%) non-Beijing strains. The proportion of PZA-resistant isolates was similar between the Beijing and non-Beijing strains.

**Table 1:** Demographic data of the multidrug-resistant tuberculosis patients with or without pyrazinamide (PZA) resistance^a^

	PZA susceptibility test		P value
		
	Resistant, n = 36	Susceptible, n = 30	
Age (MD)	58.3 (21.0)	57.8 (17.8)	0.91
Gender			
Male	25 (69.4%)	24 (80%)	0.33
Female	11 (30.6%)	6 (20%)	
Previous anti-TB treatment			
Yes	11 (30.6%)	10 (33.3%)	0.81
No	25 (69.4%)	20 (66.7%)	
Sputum smear			
Positive	17 (47.2%)	22 (73.3%)	0.032
Negative	19 (52.8%)	8 (26.7%)	
Disease site			
Pulmonary TB	35 (97.2%)	28 (93.3%)	0.45
Extrapulmonary TB	1 (2.8%)	2 (6.7%)	
Presence of cavity in radiogram			
Yes	8 (22.2%)	12 (40%)	0.12
No	28 (77.8%)	18 (60%)	
Ethambutol resistance			
Yes	21 (58.3%)	10 (33.3%)	0.043
No	15 (41.7%)	20 (66.7%)	
Streptomycin resistance			
Yes	24 (66.7%)	11 (36.7%)	0.015
No	12 (33.3%)	19 (63.3%)	
Genotyping			
Beijing strain	21 (58.3%)	16 (53.3%)	0.68
Non-Beijing strain	15 (41.7%)	14 (46.7%)	

^a^Data are presented as n(%) unless otherwise stated.

### PZA susceptibility results and mutations in the *pncA *gene

The mutations in the *pncA *gene and the correlation with PZA susceptibility testing are shown in Table 2. Of 66 clinical MDRTB isolates, 36 were wild type and the other 30 were associated with at least one *pncA *gene mutation. Among these 30 isolates, we found 36 unique mutations out of a total of 39 different mutations. The majority of these were point mutations that resulted in nucleotide substitutions (37/39, 94.9%). There was one deletion (1/39, 2.6%) and one insertion (1/39, 2.6%). In addition, three isolates were identified to possess more than one site of *pncA *mutation in a single isolate, and all of these isolates were resistant to PZA. The mutations were scattered along the *pncA *gene and no highly conservative region could be identified. Seven isolates with the wild-type *pncA *gene were found to be resistant to PZA. Meanwhile, one isolate which was susceptible to PZA had a *pncA *gene mutation (A98C, Asp(33)→Ala).

**Table 2:** *pncA *gene mutation analysis, PZase activity test, and spligotyping of the MDRTB isolates

Mutation site	No. of isolates	Nucleotide substitution	Amino acid change	PZA susceptibility	PZase activity	Spoligotyping
7	1	G→T	Ala(3)→Ser	Resistant	+	Beijing
68	1	G→C	Gly(23)→Ala	Resistant	+	H3
83	1	C→T	Ala(28)→Val	Resistant	+	T1
98	1	A→C	Asp(33)→Ala	Sensitive	+	Beijing
134	1	T→G	Val(45)→Gly	Resistant	-	Beijing
135	1	T→C	Val(45)→Ala	Resistant	-	Orphan strain
161	1	C→T	Pro(54)→Leu	Resistant	+	Orphan strain
165	1^a^	T→A	Gly(55)→Gly	Resistant	-	Beijing
166	1^a^	G→T	Gly(55)→Gly	Resistant	-	Beijing
167	1^a^	A→G	Asp(56)→Cys	Resistant	-	Beijing
170	1^a^	A→C	His(57)→Pro	Resistant	-	Beijing
171	1, 1^a^	C→T	His(57)→Pro	Resistant	1+, 1-	Beijing
174	1^a^	C→A	Phe(58)→Leu	Resistant	-	Beijing
168	1	Ins C	Frameshift	Resistant	-	Beijing
185	1	C→A	Pro(62)→Gln	Resistant	+	Beijing
211	1	C→T	His(71)→Tyr	Resistant	-	Beijing
225	1	A→G	Thr(76)→Ala	Resistant	-	Orphan strain
233	1	G→T	Gly(78)→Gly	Resistant	-	Beijing
248	1	C→G	Pro(83)→Arg	Resistant	+	Orphan strain
254	1	T→C	Leu(85)→Pro	Resistant	-	Beijing
290	1	G→A	Gly(97)→Asp	Resistant	-	Beijing
298	1^b^	A→G	Thr(100)→Ala	Resistant	-	Orphan strain
442	1^b^	C→T	Arg(148)→Cys	Resistant	-	Orphan strain
308	1	A→G	Tyr(103)→Cys	Resistant	-	Beijing
207-209	1^c^	del ACC	-	Resistant	-	Orphan strain
319	1^c^	G→T	Glu(107)→Glu	Resistant	-	Orphan strain
403	1^c^	A→G	Thr(135)→Ala	Resistant	-	Orphan strain
461	1^c^	G→C	Arg(154)→Thr	Resistant	-	Orphan strain
335	2	A→T	Asn(112)→Tyr	Resistant	1+, 1-	Beijing, T2
364	1	C→T	Gln(122)→Gln	Resistant	-	Beijing
394	1	G→A	Gly(132)→Ser	Resistant	+	Orphan strain
395	1	G→A	Gly(132)→Asp	Resistant	-	Orphan strain
401	1	C→T	Ala(134)→Val	Resistant	-	Beijing
416	2	T→C	Val(139)→Ala	Resistant	1+, 1-	Beijing, U
529	1	A→C	Thr(177)→Pro	Resistant	+	Orphan strain
551	1	G→A	Csy(184)→Tyr	Resistant	-	Beijing
-	7	WT	WT	Resistant	4+, 3-	Beijing, Orphan strain
-	29	WT	WT	Sensitive	27+, 2-	Beijing, H3, Orphan strain, T1, T2-T3

^a, b, c ^Multiple mutations within one isolate

WT: wild type

### Genotyping and PZA susceptibility results

The comparison of PZA susceptibility testing and *pncA *mutations and the impact of genotyping are shown in Table 3. Among 36 isolates with PZA resistance, 29 were documented with *pncA *gene mutations. Among 30 isolates which were susceptible to PZA, 29 were associated with the wild type *pncA *gene. Overall, the sensitivity and specificity of *pncA *gene mutations in predicting PZA resistance were 80.6% and 96.7%, respectively. In isolates belonging to Beijing genotypes (37 isolates), the sensitivity and specificity were 76.2% and 93.8%, respectively. In non-Beijing strains (29 isolates), the sensitivity and specificity were 86.7% and 100%, respectively.

**Table 3:** Concordance of *pncA *gene analysis and PZA susceptibility testing by the BACTEC MGIT 960 method

	*pncA *gene mutation		Sensitivity (%)	Specificity (%)	PPV (%)	NPV (%)
					
PZA susceptibility	Yes					
Overall, n = 66			80.6	96.7	96.7	80.6
Resistance	29	7				
Susceptible	1	29				
Beijing strain, n = 37			76.2	93.8	94.1	75
Resistance	16	5				
Susceptible	1	15				
Non-Beijing strain, n = 29			86.7	100	100	87.5
Resistance	13	2				
Susceptible	0	14				

PZA, pyrazinamide; PPV, positive predictive value; NPV, negative predictive value.

The concordance of PZA susceptibility testing and PZase activity and the impact of genotyping are shown in Table 4. Among 36 PZA-resistant isolates, 21 had no detectable PZase activity as judged by Wayne's method. In 30 PZA-sensitive isolates, 28 had positive PZase activity. The overall sensitivity and specificity of PZase activity testing in predicting PZA resistance were 58.3% and 93.3%, respectively. In 37 Beijing strain isolates, the sensitivity and specificity were 66.7% and 93.8%, respectively. In 29 non-Beijing strain isolates, the sensitivity and specificity were 46.7% and 92.9%, respectively.

**Table 4:** Concordance of PZase activity and PZA susceptibility testing by the BACTEC MGIT 960 method

	PZase activity		Sensitivity (%)	Specificity (%)	PPV (%)	NPV (%)
					
PZA susceptibility	Negative					
*Overall, n = 66*			58.3	93.3	91.3	65.1
Resistance	21	15				
Susceptible	2	28				
Beijing strain, n = 37			66.7	93.8	93.3	68.2
Resistance	14	7				
Susceptible	1	15				
Non-Beijing strain, n = 29			46.7	92.9	87.5	61.9
Resistance	7	8				
Susceptible	1	13				

PZase, pyrazinamidase; PZA, pyrazinamide; PPV, positive predictive value; NPV, negative predictive value.

## Discussion

PZA is an important and effective first-line anti-TB drug both in MDRTB and fully susceptible isolates. However, traditional susceptibility testing of PZA is of limited value and not routinely performed. Detection of *pncA *gene mutations may provide rapid and reliable information about PZA susceptibility profiles. In our analysis, more than half of the MDRTB isolates were also resistant to PZA. No clinical characteristics have a good correlation with PZA resistance, except for concomitant resistance to other first-line anti-TB agents. The analysis of *pncA *gene mutations is a test of high specificity and moderate sensitivity in predicting PZA resistance. The correlation between *pncA *gene mutations and PZA resistance is slightly better in non-Beijing strains as compared with Beijing strains. The present study suggests that *pncA *gene analysis is useful in identifying PZA susceptibility profiles in MDRTB isolates, especially in non-Beijing strains. However, the presence of wild type *pncA *genes in PZA-resistant isolates also suggests that mechanisms other than *pncA *gene mutations leading to PZA resistance may exist, and this deserves further exploration.

Classified as a first-line oral anti-TB agent, PZA is widely used in the intensive phase of anti-TB treatment and plays a pivotal role in the treatment regimen of MDRTB and extensively drug-resistant tuberculosis (XDRTB) disease. Unfortunately, concomitant resistance to other first-line anti-TB drugs, including PZA, is not uncommon in MDRTB and XDRTB isolates. Due to difficulties in performing PZA susceptibility tests and the unreliable results of the traditional method, information about PZA resistance in MDRTB isolates is not routinely obtained in clinical settings. Recent studies from South Africa and Thailand reported a PZA resistance rate of around 50% in MDRTB isolates (24, 25). In line with these reports, the PZA resistance rate was 54.5% in the present study by the BACTEC MGIT 960 method. The underlying mechanism leading to the high PZA resistance rate in MDRTB isolates remains to be identified. However, an increased incidence of previous PZA exposure in these isolates is a possible cause (26). Assuming that approximately half of MDRTB isolates are resistant to PZA, then nearly half of them are PZA-susceptible. If we can identify the PZA-susceptible MDRTB isolates timely and accurately, it would be possible to add PZA to the combination of anti-TB drugs for MDRTB patients.

The early identification of drug susceptibility by drug resistance gene analysis has been widely used for first-line anti-TB drugs in recent years, especially rifampicin and isoniazid (27). The PZase enzyme, encoded by the *pncA *gene, plays a vital role in the bactericidal effect of PZA against MTB isolates (11-13). Theoretically, *pncA *gene analysis should enable clinicians to identify PZA resistance early. However, the correlation between *pncA *gene mutations and PZA susceptibility varies among previous studies (14-17). As compared with the BACTEC MGIT 960 method, the overall sensitivity and specificity of *pncA *gene analysis in the present study were 80.6% and 96.7%, respectively, which is comparable with previous reports (17, 24). The high specificity suggests that *pncA *gene analysis is an ideal method to identify PZA-resistant MDRTB isolates. Meanwhile, the relatively lower sensitivity means that false negative results are possible and implies that mechanisms other than *pncA *mutations are involved in PZA resistance. Although *pncA *gene analysis is a rapid test with a high positive predictive value, it cannot completely replace the phenotypic susceptibility testing of PZA.

We found a high diversity of *pncA *gene mutations without major hot spots in the MDRTB isolates. Among 36 PZA-resistant MDRTB isolates, 38 different mutations scattered along the whole *pncA *gene were identified out of 29 isolates. Meanwhile, one mutation at nucleotide 98(A→C) was found in one isolate among 30 PZA-susceptible MDRTB isolates. No clustered isolates were found to share identical *pncA *gene mutations and spoligotyping in our analysis. Our findings of scattered mutations support similar observations in previous studies (16, 24). The high diversity of *pncA *gene mutations could also be used as a supplement to current genotyping methods in epidemiological investigations.

In the present study, we also evaluated PZase activities by Wayne's test, which is based on the detection of POA. The specificity of PZase activity in predicting PZA susceptibility was comparable with that of *pncA *gene analysis, but the sensitivity was much lower. Moreover, a significant proportion of isolates with *pncA *gene mutations possessed detectable PZase activity (14/30, 46.7%). According to previous reports, the sensitivity of the PZase assay ranged from 79% to 96% and the specificity was generally above 95% (28, 29). The cause of the remarkable discordance between the PZase assay test, *pncA *gene analysis, and PZA susceptibility in the present study could not be readily clarified in the study design. The geographic differences in MDRTB isolates may have contributed to the differences between our findings and previous reports. It is possible that some *pncA *gene mutations may lead to a reduction, but not loss, of PZase activity that can still be detected by Wayne's test. Although the PZase activity is not totally lost in these isolates, it results in phenotypical resistance to PZA. Unlike previous studies that enrolled both MDRTB and non-MDRTB isolates, only MDRTB isolates were included for analysis in the present study. The different composition of MTB isolates, including various genotypes and drug susceptibility profiles, may affect the predictive value of PZase activity testing and causes of the discordant results.

The difference of PZA susceptibility and *pncA gene *mutations among various genotypes of MTB isolates has rarely been evaluated before. The Beijing strain is the dominant strain in Eastern Europe and Southeastern Asia, including Taiwan (30). Differences in clinical presentations and treatment outcomes have been reported, although with some controversy among various geographical areas (3, 31). In the present study, we compared the correlation of *pncA *gene analysis, PZase activity, and PZA susceptibility between Beijing and non-Beijing strains. We demonstrated that the Beijing strain had a slightly lower sensitivity in *pncA *gene analysis, but much higher sensitivity in PZase activity test, as compared with non-Beijing strains. By comparison, the specificity was similar between Beijing and non-Beijing strains in both *pncA *gene analysis and PZase activity tests. No specific mutation was found in Beijing or non-Beijing genotype isolates. Our data demonstrated different genetic and phenotypic characteristics of Beijing and non-Beijing strains in PZA resistance. The differences between Beijing and non-Beijing strains in PZA susceptibility warrant further verification with a larger sample size, and the underlying mechanism remains to be clarified.

In the present study, we found that MDRTB patient with concomitant PZA resistance were more likely to be sputum smear negative. The cause of significant correlation between PZA susceptibility and sputum smear results cannot be easily identified in the study design. A recent study showed that exposure of lowly active MTB bacilli to anti-TB agents led to the emergence of resistant mutants and it required higher drug concentrations to eliminate metabolic inactive mycobacteria (32). Therefore, the MDRTB isolates with PZA resistance are probably less metabolic active which may lead to the lower sputum smear positive rate. Further in-vivo and in-vitro studies will be needed to verify the issue and elucidate the underlying mechanism.

Our study has several limitations. Only MDRTB isolates were included for analysis and the predictive value of *pncA *gene analysis in non-MDRTB isolates could not be evaluated in the present study. We did not evaluate the mutation of other important drug resistant gene, such as *rpoB, inhA*, and *katG*, in these isolates. The relatively small case numbers may also lead to a lower power of our analysis. More importantly, this study was performed in a TB endemic area with a predominance of the Beijing strain. It remains to be determined if our findings can be applied to areas with a lower TB incidence or without predominance of the Beijing strain.

## Conclusions

Our study demonstrated that the PZA resistant rate in MDRTB isolates was high, and the majority of the PZA-susceptible isolates presented with wild type *pncA *genes. Analysis of *pncA *genes is a test of high specificity in predicting PZA resistance, enabling clinicians to add PZA timely and accurately to treatment regimens for MDRTB patients. The concordance between *pncA *gene analysis and PZA resistance was better in non-Beijing as compared with Beijing strains, suggesting that *pncA *gene analysis is more suitable for MDRTB isolates belonging to non-Beijing strains.

## Competing interests

The authors declare that they have no competing interests.

## Authors' contributions

YCC conceived of the study, participated in its design, reviewed the medical records, performed the statistical analysis, and drafted the manuscript. SFH and JYF helped to review the medical records and draft the manuscript. KWY, YCL and WJS participated in the design of the study and coordination. WJS carried out the genotyping studies. All authors read and approved the final manuscript.

## Pre-publication history

The pre-publication history for this paper can be accessed here:

http://www.biomedcentral.com/1471-2334/11/240/prepub
